# Modulations in neural pathways excitability post transcutaneous spinal cord stimulation among individuals with spinal cord injury: a systematic review

**DOI:** 10.3389/fnins.2024.1372222

**Published:** 2024-03-25

**Authors:** Shirin Tajali, Gustavo Balbinot, Maureen Pakosh, Dimitry G. Sayenko, Jose Zariffa, Kei Masani

**Affiliations:** ^1^KITE Research Institute – University Health Network, Toronto, ON, Canada; ^2^Krembil Research Institute, University Health Network, Toronto, ON, Canada; ^3^Center for Advancing Neurotechnological Innovation to Application – CRANIA, University Health Network, Toronto, ON, Canada; ^4^Library & Information Services, University Health Network, Toronto Rehabilitation Institute, ON, Canada; ^5^Department of Neurosurgery, Center for Neuroregeneration, Houston Methodist Research Institute, Houston, TX, United States; ^6^Edward S. Rogers Sr. Department of Electrical and Computer Engineering, University of Toronto, Toronto, ON, Canada; ^7^Institute of Biomedical Engineering, University of Toronto, Toronto, ON, Canada; ^8^Rehabilitation Sciences Institute, University of Toronto, Toronto, ON, Canada

**Keywords:** transcutaneous spinal cord stimulation, spinal cord injury, neuroplasticity, spinal excitability, supraspinal excitability

## Abstract

**Introduction:**

Transcutaneous spinal cord stimulation (TSCS), a non-invasive form of spinal cord stimulation, has been shown to improve motor function in individuals living with spinal cord injury (SCI). However, the effects of different types of TSCS currents including direct current (DC-TSCS), alternating current (AC-TSCS), and spinal paired stimulation on the excitability of neural pathways have not been systematically investigated. The objective of this systematic review was to determine the effects of TSCS on the excitability of neural pathways in adults with non-progressive SCI at any level.

**Methods:**

The following databases were searched from their inception until June 2022: MEDLINE ALL, Embase, Web of Science, Cochrane Library, and clinical trials. A total of 4,431 abstracts were screened, and 23 articles were included.

**Results:**

Nineteen studies used TSCS at the thoracolumbar enlargement for lower limb rehabilitation (gait & balance) and four studies used cervical TSCS for upper limb rehabilitation. Sixteen studies measured spinal excitability by reporting different outcomes including Hoffmann reflex (H-reflex), flexion reflex excitability, spinal motor evoked potentials (SMEPs), cervicomedullay evoked potentials (CMEPs), and cutaneous-input-evoked muscle response. Seven studies measured corticospinal excitability using motor evoked potentials (MEPs) induced by transcranial magnetic stimulation (TMS), and one study measured somatosensory evoked potentials (SSEPs) following TSCS. Our findings indicated a decrease in the amplitude of H-reflex and long latency flexion reflex following AC-TSCS, alongside an increase in the amplitudes of SMEPs and CMEPs. Moreover, the application of the TSCS-TMS paired associative technique resulted in spinal reflex inhibition, manifested by reduced amplitudes in both the H-reflex and flexion reflex arc. In terms of corticospinal excitability, findings from 5 studies demonstrated an increase in the amplitude of MEPs linked to lower limb muscles following DC-TSCS, in addition to paired associative stimulation involving repetitive TMS on the brain and DC-TSCS on the spine. There was an observed improvement in the latency of SSEPs in a single study. Notably, the overall quality of evidence, assessed by the modified Downs and Black Quality assessment, was deemed poor.

**Discussion:**

This review unveils the systematic evidence supporting the potential of TSCS in reshaping both spinal and supraspinal neuronal circuitries post-SCI. Yet, it underscores the critical necessity for more rigorous, high-quality investigations.

## 1 Introduction

Spinal cord injury (SCI) is a highly debilitating disease condition that disrupts the transmission of motor and sensory information through the spine leading to different degrees of sensorimotor impairments (Richards et al., 2017; Perrouin-Verbe et al., 2021). Based on the World Health Organization report, 250,000–500,000 new SCI cases have been identified annually (Thietje and Hirschfeld, 2017). While some sensorimotor recovery is expected within the first year of injury, less than 2% of individuals with motor complete SCI will change to incomplete SCI between the first to the fifth year of injury indicating a low probability of spontaneous recovery in the chronic phase (Steeves et al., 2011; Nardone et al., 2013). Evidence from recent clinical studies indicates that neuromodulation approaches have shown significant promise to enhance neuroplasticity and promote the recovery of motor skills after SCI (Sasmita et al., 2018; Zheng et al., 2020; Kazim et al., 2021; Zhang H. et al., 2021).

In recent times, transcutaneous spinal cord stimulation (TSCS), a non-invasive method of spinal stimulation, has emerged as a successful neuromodulation technique for enhancing motor function post-SCI (Megía García et al., 2020; Taylor et al., 2021; Rehman et al., 2023). TSCS is generally divided into two different categories: direct current (DC-TSCS) and alternating current (AC-TSCS) (Taylor et al., 2021; Barss et al., 2022; Rahman et al., 2022). In DC-TSCS (also called transspinal direct current stimulation: tsDCS), a constant electrical current, usually in the range of 1–2.5 mA, flows in a constant direction and the polarity of stimulation is defined based on the polarity of the electrode placed on the spine (cathodal or anodal) (Grecco et al., 2015; Rahman et al., 2022). However, in AC-TSCS the direction of flow and amount of electricity change cyclically over time between cathode and anode (Grecco et al., 2015; Barss et al., 2022). In AC-TSCS, a range of currents at a frequency of 0.2–50 Hz with different intensities (10–200 mA) embedded in a carrier frequency (5–10 kHz) can be used (Grecco et al., 2015; Rahman et al., 2022).

Findings from recent studies indicate that both types of TSCS currents (DC & AC) alone or in combination with activity-based rehabilitation programs can improve motor function after SCI (Hubli et al., 2013; Abualait and Ibrahim, 2020; Megía García et al., 2020; Taylor et al., 2021; Laskin et al., 2022; Alashram et al., 2023). Specifically, the application of TSCS led to a decrease in the need for external assistance in the upright stance, a decrease in spasticity, and an increase in walking speed, handgrip strength, pinch, and manual dexterity (Powell et al., 2016; Inanici et al., 2018; Abualait and Ibrahim, 2020; Alashram et al., 2023). However, the majority of studies aimed at improving motor function after SCI have used AC-TSCS (Megía García et al., 2020; Taylor et al., 2021; Barss et al., 2022; Laskin et al., 2022). As a result, several reviews have attempted to summarize the stimulation protocols and electrode parameters of studies that used AC-TSCS to improve upper and lower-extremity motor functions by measuring performance-based tests, clinical muscle strength tests, and surface electromyography recordings (Megía García et al., 2020; Taylor et al., 2021; Barss et al., 2022; Laskin et al., 2022; Rehman et al., 2023). In 2020, Megía García et al. (2020) published a systematic review of 15 studies that investigated the therapeutic effects of AC-TSCS on voluntary motor response in the SCI population, however, given the low methodological quality no definite conclusions were drawn regarding its effectiveness. Later, Taylor et al. systematically classified studies that employed TSCS in the SCI population into two different categories: (1) studies that used TSCS for neurophysiological investigation, i.e., measurements of spinal motor evoked potential (SMEPS) using pulse AC-TSCS, and (2) studies that used continuous AC-TSCS as a therapeutic modality for improving motor function (muscle force, joint angle, and gait performance) (Taylor et al., 2021). In a narrative review, Barss et al. (2022) discussed that the application of AC-TSCS over multiple segments can facilitate spinal and corticospinal excitability in neurologically intact individuals and those with SCI. Recently, in a scoping review, Rehman et al. (2023) reported some neural mechanisms that underlie TSCS to enable motor function as well as details about TSCS settings (electrodes, amplitude, frequency, and shape of stimulation). However, their report regarding the mechanism of action was not exclusive to the SCI population and only included six studies. So far, there has not been a comprehensive investigation that systematically explores changes in the spinal and supraspinal mechanisms following different types of TSCS currents (DC-TSCS & AC-TSCS) that might influence the recovery process after SCI (Barss et al., 2022). We believe that a more accurate understanding of possible neuromodulation in the spinal and supraspinal pathways will improve the design and execution of TSCS protocols, yielding more robust clinical benefits.

The results of recent computer modeling, preclinical and neurophysiological studies have provided evidence that TSCS can recruit low threshold, large-to-medium diameter afferents within the dorsal column of the spinal cord, which, in turn, can activate motor neurons involved in the regulation of movement (Sdrulla et al., 2018; Filipp et al., 2019; Kazim et al., 2021; Taylor et al., 2021). Several recent clinical studies in the human SCI and neurologically intact populations have revealed that TSCS interventions can modulate spinal and/or cortical networks controlling the muscles after the intervention (Hubli et al., 2013; Bocci et al., 2015; Powell et al., 2016, 2018; Abualait and Ibrahim, 2020; Benavides et al., 2020; Hofstoetter et al., 2020; Al’joboori et al., 2021; Kaneko et al., 2021; Kazim et al., 2021; Barss et al., 2022). Studies investigating the excitability of spinal networks mainly employed the Hoffmann reflex (H-reflex), tibialis anterior (TA) flexion reflex arc, and SMEPs as outcomes (Hubli et al., 2013; Knikou and Murray, 2019; Murray and Knikou, 2019b). The H-reflex is a monosynaptic spinal reflex response obtained from stimulation of the afferent peripheral nerve, while the TA flexion reflex is a polysynaptic reflex typically elicited by electrical stimulation of the distal tibial nerve (Pulverenti et al., 2021, 2022). SMEPs (also called posterior root-muscle reflex, multi-segmental monosynaptic response, or transspinal evoked potential] are multi-segmental muscle responses evoked by the stimulation of dorsal roots which eventually activate motoneurons in the spinal cord (Taylor et al., 2021). For example, Knikou et al. investigated the effects of AC-TSCS on soleus H-reflex excitability in individuals living with and without SCI through case–control studies and found that H-reflex excitability decreased after training with TSCS in individuals with SCI indicating changes in the functional connectivity within the spinal neural networks after training (Knikou and Murray, 2019). Hubli et al. (2013) found increased spinal reflex amplitude as measured by TA flexion reflex after a session of anodal DC-TSCS combined with locomotion in individuals with motor complete SCI (Hubli et al., 2013). Several recent pre-post interventional studies have shown that the application of cervical AC-TSCS can increase the amplitude of SMEPs in arm and hand muscles (Gad et al., 2018; Inanici et al., 2018; Zhang et al., 2020).

Nonetheless, to date, no comprehensive review has thoroughly assessed the collective findings from studies examining modulation in neural pathway excitability subsequent to different types of TSCS among adults with SCI. Hence, this systematic review aimed to consolidate the available evidence concerning the impact of TSCS interventions on the excitability of both spinal and supraspinal pathways in individuals living with SCI. Our focus encompassed a detailed exploration of various TSCS interventions—AC-TSCS and DC-TSCS—and their effects on neurophysiological variables measuring excitability within the spinal (e.g., H-reflex, flexion reflex arc excitability, SMEPs) and corticospinal pathways (MEPs induced by TMS) subsequent to TSCS.

## 2 Methods

### 2.1 Registry of systematic review protocol

The protocol of this systematic review was registered and published on PROSPERO in September 2022.^1^

### 2.2 Information sources

The following databases were searched from their inception until June 2022: MEDLINE ALL, Embase, Web of Science, Cochrane Library, and clinical trials. The search was performed with the help of a research librarian (MP) and the following MeSH terms were used: “transcutaneous spinal cord stimulation” OR “TSCS” OR “TSS” OR “TSDCS” OR “transspinal stimulation” AND “corticospinal excitability” OR “neuroplasticity” OR “cortical motor evoked potential” OR “spinal motor evoked potential” OR “spinal reflex” OR “H-reflex” OR “F-wave” OR “recruitment curve” AND “spinal cord injury.” Data were extracted from the following databases and were imported into Covidence, a management software for systematic reviews (Covidence, Melbourne, Australia). Abstract screening and full-text screening were all completed in Covidence.

### 2.3 Eligibility criteria

The eligibility criteria were designed based on the PICO model: Population: “Spinal Cord Injury,” Intervention/Identifier: “TSCS,” Comparators: no intervention, sham intervention, or pre-post analysis, and Outcome of interest: “neurophysiological variables.” We included studies that used any type of TSCS current including AC-TSCS, DC-TSCS, and spinal paired stimulation as the main intervention in individuals living with non-progressive SCI. In addition, the included studies needed to have reported any neurophysiological outcomes related to the excitability of neural pathways following intervention, such as H-reflex, SMEPs, or MEPs. We excluded animal studies, studies that used invasive stimulation such as epidural stimulation, studies in which the full text was not available in English, review articles, and conference proceedings. Studies were excluded in our systematic review if they did not investigate any neurophysiological outcomes following TSCS and/or did not include the SCI population.

### 2.4 Study selection

Two independent authors (S.T & G.B) performed screening and two senior authors (K.M & J.Z) resolved conflicts regarding the eligibility of studies. The screening and selection process was performed using the Covidence software.

### 2.5 Data extraction

Data including study designs, demographic and clinical characteristics of the population (age, sex, time since injury, level of injury), parameters of TSCS (vertebral level & type of stimulation, frequency, pulse width, duration, and intensity), number and durations of training sessions, time points of assessment, outcome measures related to neuroplasticity, methods of measurements and other reported outcomes were extracted.

### 2.6 Study design & quality assessment

The modified Downs and Black (D&B) Checklist with the corresponding quality levels was used to assess the quality of included articles: excellent (26–28); good (20–25); fair (15–19); and poor (≤14) (O’Connor et al., 2015). The checklist consists of 27 questions, each question is rated either as yes (=1) or no/unable to determine (=0), and one item has a 3-point scale (yes = 2, partial = 1, and no = 0). It measures the quality of the reporting (10 questions), the external validity (3 questions), the internal validity (bias and confounding: 13 questions), and the power of the study (1 question) (Hootman et al., 2011; Lee et al., 2022).

## 3 Results

### 3.1 Search findings

**Figure 1:**
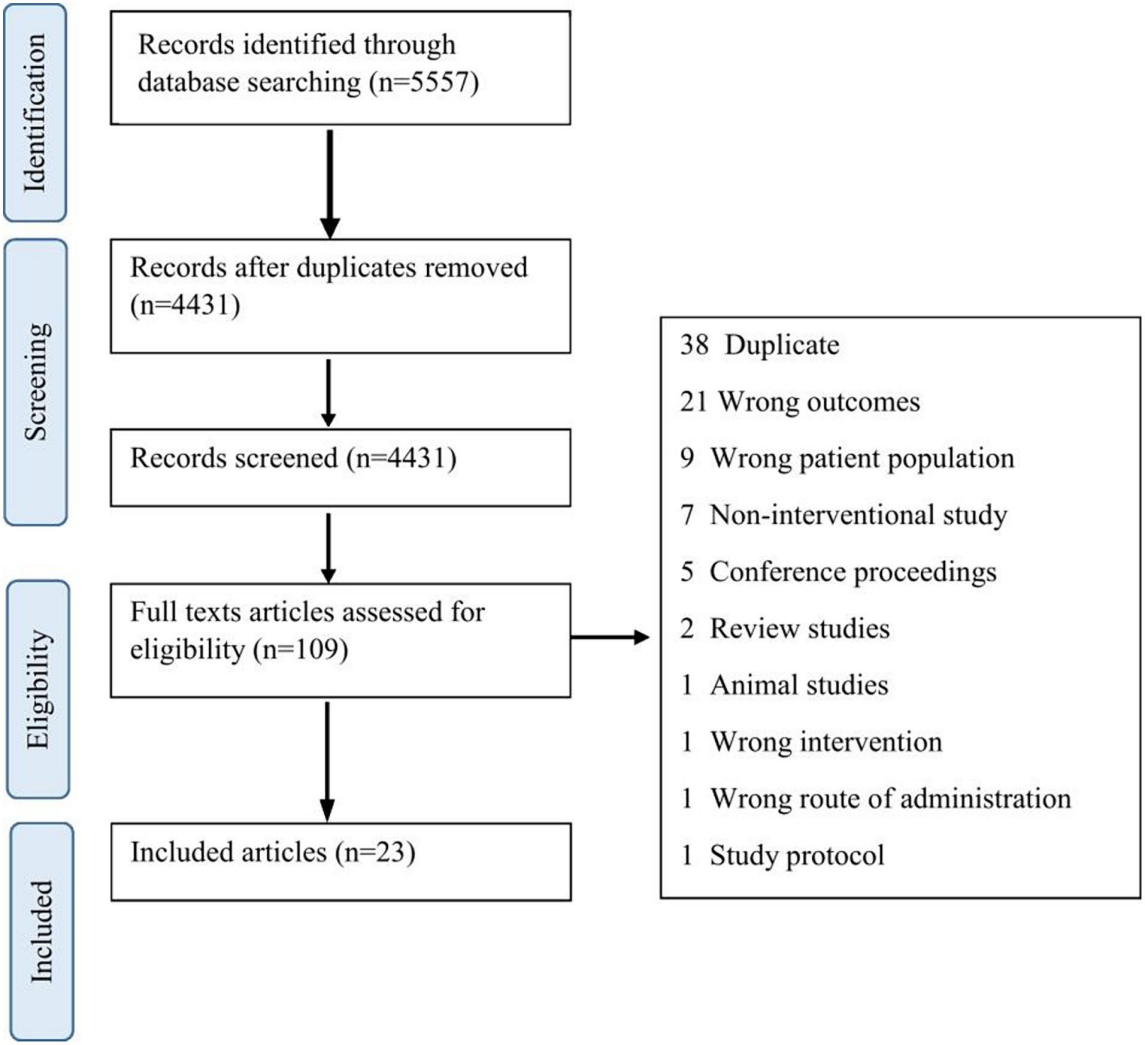
PRISMA flow diagram of the screening and selection process.

Figure 1 illustrates the Preferred Reporting Items for Systematic Review and Meta-Analysis (PRISMA) flowchart for this study. A total of 4,331 abstracts were screened, and 23 articles were included. Table 1 shows the demographics and clinical characteristics of study participants as well as different designs of studies including case studies or series (*n* = 8), single-arm pre-post interventions (*n* = 7), case–control (*n* = 5), and clinical trials (*n* = 3).

**Table 1:** Study designs and participants’ characteristics of included studies.

Author (Year)	Study design	Sample	Age	Sex	AIS	Level of injury	Time since injury	Healthy control group characteristics
Hubli et al. (2013)	Case–control study	Total 34 (17 SCI, 17 controls)	Mean (SD):35.9 (13.7) years	16 males, 1 female	AIS A (*n* = 10), AIS B (*n* = 7)	C3- T6	Mean (SD): 96.0 (78.3) months	Mean (SD) of age: 29.9 (8.1) years; 11 male, 6 female
Gerasimenko et al. (2015)	Single-arm interventional study	5 SCI	Mean (SD): 31.4 (15.0) years	5 males	AIS(B)	Above T5	More than 1 year	NA
Powell et al. (2016)	Case study with cross-over design	1 SCI	56 years	Female	AIS (D)	Motor level: L1 bilaterally, sensory levels: T2 (right) and T3 (left).	41 years	NA
Murray and Knikou (2017)	Case study	1 SCI	27 years	Male	AIS C for upper extremities and AIS B for lower extremities	C6-C7	9 years	NA
Gad et al. (2018)	Single-arm interventional study	6 SCI	age > 18 years		AIS B (*n* = 2), AIS C (*n* = 4)	Above C7	more than 1 year (range: 1–21 years)	NA
Inanici et al. (2018)	Case study	1 SCI	62 years	Male	AIS (D)	C3	2 years	NA
Powell et al. (2018)	Single-blind, sham-controlled, randomized crossover design	6 SCI	Mean (SD):15.7 (12.1) years	4 male/ 2 female	AIS C (*n* = 4), AIS D (*n* = 2)	Above L2 (1 cervical, 2 thoracic, 2 lumbar)		NA
Knikou and Murray (2019)	Case–control study	Total 20 (10 SCI, 10 healthy controls)	Mean (SD):36.3(11.1) years	7 male/3 female	AIS A (*n* = 2), AIS B (*n* = 2), AIS C (*n* = 1), AIS D (*n* = 5)	C4-T11	Mean (SD): 8.8 (8.1) years	Mean (SD) of age: 30.9 ± 14 year; 5 male, 5 female
Murray and Knikou (2019b)	Case–control study	Total 20 (10 SCI, 10 healthy controls)	Mean (SD):36.3(11.1) years	7 male/3 female	AIS A (*n* = 2), AIS B (*n* = 2), AIS C (*n* = 1), AIS D (*n* = 5)	C4 - T11	Mean (SD): 8.8 (8.1) years	Mean (SD) of age: 30.9 ± 14 year; 5 male, 5 female
Sayenko et al. (2019)	Double-blind, within-subject crossover, and sham-controlled study design	15 SCI	31.2(8.7) years	12 male, 3 female	AIS A (*n* = 11), AIS B (*n* = 1), AIS C (*n* = 3)	C4-T12	Mean (SD): 6.03 (3.2) years	NA
Abualait and Ibrahim (2020)	Double-blind, sham-controlled case report with cross-over design	2 SCI	22 and 24 years	2 male	Incomplete SCI (AIS-C)	T10-T11	2 years	NA
Benavides et al. (2020)	Case–control with cross-over design	32 total (17 SCI, 15 control)	Mean (SD): 43.1(14.0) years	13 male-4 female	AIS A (*n* = 4), AIS B (*n* = 3), AIS C (n = 2), and AIS D (*n* = 8).	C4 –C6	More than 1 year	mean(SD) of age:36.5(17.5) years; 6 females
Hofstoetter et al. (2020)	Single-session pre-post design	12 SCI	Mean (SD):41.3(19.1) years	9 male/ 3 female	AIS A (*n* = 3), AIS C (*n* = 3), AIS D (*n* = 6)	Above T7	Mean (SD):18(5.2) years	NA
Meyer et al. (2020)	Single-arm, single-session	10 SCI	Mean (SD):45.4(12.4)	9 male/1 female	AIS D (*n* = 10)	C3 -T10	Mean (SD): 11.6 (10.2) years	NA
Shapkova et al. (2020)	Clinical trial	Total 35 (group 1 SCI:19, group 2 SCI: 16)	Main SCI Group: 31.2(8.6), control SCI group 2: 33.3 (9.3) years	Main SCI group: 15 male/4 female, Control SCI group: 10 male/ 6 female	Main SCI group: AIS A (*n* = 11), AIS B (*n* = 5), AIS C (*n* = 3) /Control SCI group: AIS A (*n* = 7), AIS C (*n* = 5), AIS D (*n* = 4)	Main SCI group:15 had a lesion at thoracic level, 2 participants at thoracic-lumbar level, and 2 participants at low-cervical level. Control SCI group:12 participants had a lesion at thoracic level, 3 at thoracic-lumbar level, and 1 at low-cervical high thoracic level.	More than 1 year	NA
Zhang et al. (2020)	Case study	1 SCI	38 years	male	AIS (A)	C5	15 years	
Islam et al. (2021)	Case–control study	Total 18 (5 SCI, 13 controls)	Mean (SD):43.8 (11.4) years	four male, one female	AIS B (*n* = 1), AIS C (*n* = 1), AIS D (*n* = 3)	Above T12 (3 cervical-1 thoracic)	Mean (SD):13.4 (9.0) years	Age: 19–35 years; 5 male and 8 female
Pulverenti et al. (2021)	Randomized clinical trial	Total 14 (group 1 SCI:7, group 2 SCI:6)	Group 1: 43.3 (15.8), group 2: 47.5 (16.2) years	Group 1 SCI: 6 male/1 female, Group 2: 5 male/ 1 female	Group 1: AIS A (*n* = 1), AIS B (*n* = 1), AIS C (*n* = 3), AIS D (*n* = 3) /Group 2: AIS A (*n* = 1), AIS C (*n* = 3), AIS D (*n* = 2)	C4-T12	Group 1 mean (SD):9 (3.6), Group 2 mean (SD): 7.3 (4.6) years	NA
Zaaya et al. (2021)	Case-series	4 SCI	35.5 (8.9) years	4 male	AIS (B)	C6-T9	More than 6 months	NA
Zhang Z. et al. (2021)	Case series	3 SCI	55.3 (10.0) years	3 male	AIS C (*n* = 2), AIS D (*n* = 1)	C4-C5	Mean (SD): 4.3 (2.8) years	NA
Adeel et al. (2022)	Single-blinded controlled study	9 SCI	Mean (SD):54.22(6.11) years	2 male-7 female	Incomplete SCI (AIS B-D)	Above T10 (5 cervical & 4 thoracic)	Mean (SD): 3.22 (2.57) years	NA
Pulverenti et al. (2022)	Randomized clinical trial	Total 11 (group 1 SCI: 5, group 2 SCI:6)	Mean (SD) of 8 participants:45.81 (15.47) years	Group 1 SCI: 4 male/1 female, Group 2: 5 male/ 1 female	Group 1: AIS A (*n* = 1), AIS C (*n* = 2), AIS D (*n* = 2) /Group 2: AIS A (*n* = 1), AIS C (*n* = 3), AIS D (*n* = 2)	C4-T12	Group 1 mean (SD):8.2 (4.4), Group 2 mean (SD): 7.3 (4.6) years	NA
Samejima et al. (2022)	Case study	2 SCI	64 years	2 male	AIS D (*n* = 2)	C4 &C6	3.5 and 4.5 years	NA

Spinal cord injury (SCI), American Spinal Injury Association Impairment Scale (AIS).

### 3.2 Changes in the excitability of spinal pathways following TSCS

**Figure 2:**
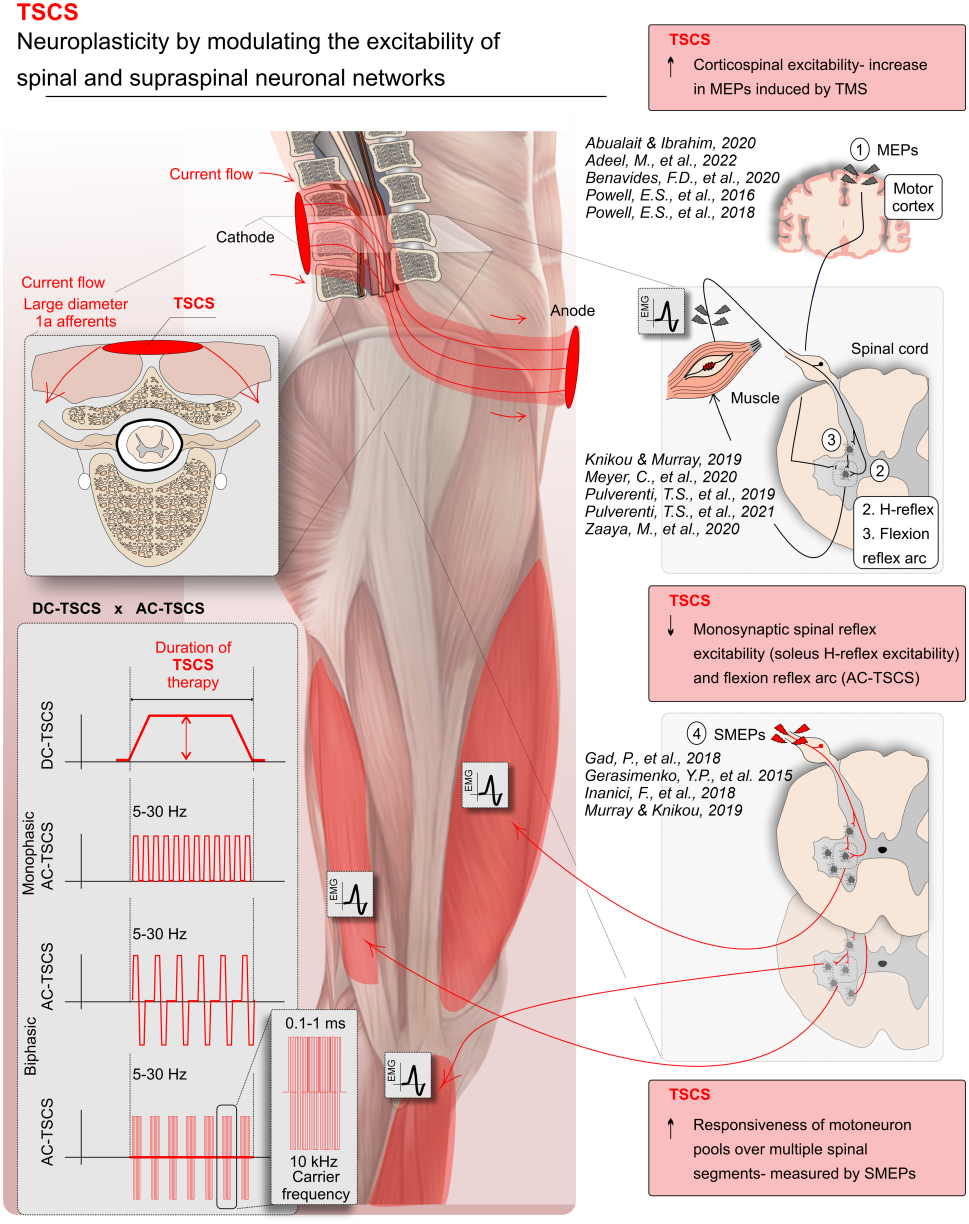
Modulations in neural pathway excitability with transcutaneous spinal cord stimulation (TSCS) in spinal cord injury (SCI). TSCS, a non-invasive form of spinal cord stimulation, utilizes electrodes on the skin to deliver direct (DC-TSCS) or alternating currents (AC-TSCS), with or without a carrier frequency. The stimulation accesses the spinal cord through the dorsal roots (the gateway to the spinal cord), targeting respective spinal segments and circuits. This systematic review predominantly draws evidence from TSCS studies, showcasing TSCS’s ability to enhance neuroplasticity by modulating spinal and supraspinal neuronal networks. Specifically, studies demonstrated increased motoneuron pool responsiveness, reflected in spinal motor evoked potentials (SMEPs) following AC-TSCS associated with improved motor output, AC-TSCS notably reduced monosynaptic spinal reflex excitability (soleus H-reflex) and flexion reflex arc. TSCS modulated corticospinal excitability, evidenced by increased motor evoked potentials (MEPs) induced by transcranial magnetic stimulation (TMS). Note that some of the depicted references in the figure come from upper limb studies.

Figure 2 provides a visual summary of the main research findings related to the effects of TSCS on the excitability of spinal and supraspinal pathways after SCI. Sixteen studies measured spinal excitability including H-reflex, flexion reflex arc excitability, SMEPs, cervicomedullary evoked potentials (CMEPs), and cutaneous-input-evoked response. Of the included studies, 2 reported changes following DC-TSCS (Hubli et al., 2013; Adeel et al., 2022), and 14 studies reported changes following AC-TSCS (Gerasimenko et al., 2015; Gad et al., 2018; Inanici et al., 2018; Knikou and Murray, 2019; Murray and Knikou, 2019a; Sayenko et al., 2019; Benavides et al., 2020; Hofstoetter et al., 2020; Meyer et al., 2020; Shapkova et al., 2020; Zaaya et al., 2020; Zhang et al., 2020; Islam et al., 2021; Pulverenti et al., 2021, 2022). Results from spinal reflexes are shown in detail in Table 2 and are summarized below. We discerned the findings of these two transcutaneous stimulation modalities (DC-TSCS&AC-TSCS) because of possible differences in the hypothesized mechanism of these two modalities in altering the excitability of neural pathways (Rahman et al., 2022; Alashram et al., 2023).

**Table 2:** Study characteristics including details of TSCS and study outcomes.

Author (Year)	Location	Type of TSCS	Frequency & pulse width	Intensity	Other interventions	comparison	Number & duration of sessions	Time points of measurements	Neurophysiological outcomes	Results of neurophysiological outcomes	Results of other outcomes
Hubli et al. (2013)	Active electrode: T11-T12, Reference electrode: left shoulder	DC-TSCS	NA	2.5 mA	In individuals with SCI: BWS walking (70%), healthy subjects:20 min walking at normal speed	4 conditions: anodal, cathodal, and sham	20 min for each session	Before (B), immediately after (t0), and 20 min after t0	TA flexion reflex (amplitude, threshold) and latency by electrical stimulation of the left tibial nerve	Anodal DC-TSCS reduced the reflex threshold and increased the reflex amplitude in SCI subjects	NA
Gerasimenko et al. (2015)	Cathode: T11 -T12 and/or over coccyx 1 (Co1), Anodes: iliac crests	AC-TSCS with a carrier frequency of 10 kHz	Monophasic (1 msec duration)	At a sub-motor threshold level	TSCS at T11, Co1 (coccyx 1), and T11 + Co1/active and passive movement/ buspirone 7.5 mg orally twice daily for the last 4 weeks	NA	18 weeks of weekly interventions (each session: 45 min)	Pre-Train:t1; post-Train: t2;pre-drug:t3, post-drug:t4	SMEPs amplitude and recruitment curve during T11 stimulation in a supine position	SMEPs were higher at t4 than t1. A dramatic increase in the amplitude of the early response at t4, as compared with t1	AIS motor scores increased at t1, t2, and t4. The mean amplitudes of hip and knee angular movements were greater t4 than Pre-t1
Powell et al. (2016)	Active electrode: T10, Reference electrode: left deltoid	DC-TSCS	Ramped up/down over a period of 30	2.5 mA for a total of 20 min	30–40 min of BWS walking immediately after DC-TSCS	Sham stimulation: ramp up/down over a 30 s	24 sessions of DC-TSCS and 24 sessions of sham stimulation with locomotor training	At baseline (t0), post-sham (t1), and post-cathodal (t2)	5 MEPs at each TMS intensity ranging from 60–100% of maximum stimulator output. Spinal excitability was measured by bilateral H reflex response in the soleus muscles	After cathodal DC-TSCS, a clear increase compared to both baseline and post-sham (ie, t2-t0 and t2-t1 comparisons) was seen on the right brain while the left brain showed an increase compared with post-sham (t2–1 comparison). No H reflexes were evoked from the subject	Improvement in 10MWT speed, SCIM-III mobility, and BBS was seen in both conditions. 6MWT worsened after sham but improved after cathodal DC-TSCS. MMT scores for lower extremities improved following sham but decreased following cathodal DC-TSCS.
Murray and Knikou (2017)	Cathode: C5, Anodes: clavicles	AC-TSCS	0.2 Hz daily	An average intensity of 42.5 mA.	NA	NA	14 sessions of AC-TSCS	Pre-post	MEPs from the right flexor and extensor carpi radialis in response to paired TMS.	The latencies of MEPs decreased, while the amplitude of MEPs increased.	Penn Spasm Frequency Scale, and ankle clonus decreased from slight to no resistance
Gad et al. (2018)	Cathodes: C3-C4 and C6-C7, Anodes: iliac crests	AC-TSCS with a carrier frequency of 10 kHz	Biphasic or monophasic rectangular 1 msec pulses at a frequency of 30 Hz	Adjusted to enable maximal grip strength (10–250 mA)	Hand grip exercises with/without stimulation		4-week intervention program (2 sessions/week, each lasting 1–2 h)	Pre, during and post intervention	SMEPs were measured from proximal and distal upper extremity muscles (bicep brachii, flexor digitorium, and extensor digitorium) by applying TSCS, 1 Hz with a 1 msec pulse width and monophasic waveform	SMEPs were larger for distal muscles at the end of the intervention, compared with before intervention	An increase in hand grip function,sensory and motor scores in International Standards for Neurological Classification of Spinal Cord Injury examination post intervention
Inanici et al. (2018)	Cathodes: C3-4 and C6-7, anodes: iliac crests	AC-TSCS with a carrier frequency of 10 kHz	Biphasic, rectangular, 1 ms pulses at a frequency of 30 Hz	80-120 mA	TSCS combined with ABT targeting upper extremity functions (first 4 weeks), (2) ABT only(second 4 weeks), and (3) TSCS+ABT for 1 week	NA	2-h sessions, 4–5 days/week, over the 9 weeks of intervention	SMEPs were recorded at the end of each week of stimulation + ABT sessions. Pinch strength was performed weekly. GRASSP tests were repeated in the first, second and fourth weeks, and once at the end of the second stimulation + ABT phase.	SMEPs were collected from deltoid, triceps, biceps, brachioradialis,extensor digitorum, flexor digitorum, abductor digiti minimi, and thenar muscles by monophasic, rectangular, 1 ms single pulses filled with a 10 kHz waveform over the C3-4	The polysynaptic, late EMG responses increased gradually over 4 weeks of stimulation combined with physical therapy, reduced after physical therapy only, but returned with 5 days of additional stimulation and therapy treatment	GRASSP, upper extremity motor score and pinch increased in both hands. Sensation recovered on trunk dermatomes, and overall neurologic level of injury improved from C3 to C4
Powell et al. (2018)	Active electrode: T10, Reference electrode: over the left deltoid	DC-TSCS	Ramped up/down over 30 s	2.5 mA for 20 min	NA	Sham stim: ramp up/down over 30 s	3 sessions, separated by at least 1 week	Pre-post	MEPs for the right and left Sol at 110% RMT.	Cathodal DC-TSCS induced an increase in MEPs-right Sol, contralateral to the reference electrode, and a decrease in MEPs-left Sol, ipsilateral to the reference electrode. Further, anodal DC-TSCS induced an increase in MEPs-left Sol (the magnitude of these changes did not reach significance).	NA
Knikou and Murray (2019)	Cathode: T10-L2, Anodes: iliac crests	AC-TSCS	Monophasic transspinal stimuli of 1 ms duration	At 0.2 Hz at subthreshold and suprathreshold intensities of the right soleus SMEPs.	NA	NA	Individuals with SCI: 16.6 ± 1 stimulation sessions for an average of 60 ± 2 min per session, Healthy controls:10 stimulation sessions (40 ± 0.1 min per session)	Pre- post	Homosynaptic depression following single tibial nerve stimuli, and post-activation depression following paired tibial nerve stimuli	Soleus H-reflex excitability decreased in both legs in motor incomplete and complete SCI but not in healthy control subjects. Homosynaptic depression increased in all SCI subjects and remained unaltered in healthy controls. Post-activation depression remained unaltered	The severity of spasms and ankle clonus decreased
Murray and Knikou (2019b)	Cathode: T10 to L1-L2, Anodes: iliac crests	AC-TSCS	Monophasic stimuli of 1-ms duration	At 0.2 Hz at subthreshold and suprathreshold intensities of the right soleus SMEPs.	NA	NA	Individuals with SCI: 16.6 ± 1 stimulation sessions for an average of 60 ± 2 min per session, Healthy controls: 10 stimulation sessions (40 ± 0.1 min per session)	Pre-post	Changes in SMEPs at stimulation frequencies of 0.1, 0.125, 0.2, 0.33 and 1.0 Hz, and post activation depression using paired stimuli at interstimulus intervals of 60, 100, 300, and 500 ms. SMEPs were recorded at rest from bilateral ankle and knee flexor/extensor muscles.	In control and complete SCI subjects, SMEPs increased for knee muscles, while in motor incomplete SCI subjects, they increased for both ankle and knee muscles. Homosynaptic depression decreased in the left medial gastrocnemius and remained unaltered in the remaining muscles in AIS C-D subjects. Post-activation depression remained unchanged in AIS C-D	NA
Sayenko et al. (2019)	Cathodes: T11–T12 or L1–L2, Anodes: iliac crests	AC-TSCS with a carrier frequency of 10 kHz	Monophasic, 1 msec pulses, at a frequency ranging between 0.2 and 30 Hz, with a carrier frequency of 10 kHz	Up to 150 mA	Game-based balance exercises (visual feedback training)	NA	12 sessions of stand training, 3 days per week	In each training session	Peak-to-peak SMEPs amplitude	The TSCS intensity to reach the motor threshold in the leg muscles decreased during the training period both in the sitting and standing positions	Improved standing with reduced support, increased leg muscle activity during supported standing and sit-to-stand, increased center of pressure excursions during self-initiated body-weight displacements
Abualait and Ibrahim (2020)	Active electrode: T10-T11, Reference electrode: left deltoid	DC-TSCS	NA	2.5 mA	BWS walking	Sham stimulation: ramp up over 30 s	Each arm: 30 sessions (20 min of stimulation), with 5 sessions per week for 6 weeks with a washout period of 2 weeks between each arm	Pre-post sham & cathode DC-TSCS for subject A,Pre-post sham & anodal DC-TSCS for subject B	Peak-to-peak MEPs induced by TMS with an intensity ranging from 80 to 130% of the RMT.	There was an increase in MEPs post-cathode and a decrease post-anode	Cathodal DC-TSCS increased the scores of 10 MWT, BBS, SCIM-III and decreased scores of MMT,MAS. Anodal stimulation increased scores in all measures
Benavides et al. (2020)	Cathode: C5-C6, Anodes: iliac crests	AC-TSCS with and without the 5 kHz carrier frequency	5 biphasic pulses (each pulse of 200 s duration) frequency of 30 Hz, Duration:20 min	Minimal intensity required to induce SMEPs in the biceps brachii	NA	Sham stimulation: same intensity as used in TSCS sessions but gradually decreased down to 0 in 1 min	Different sessions separated by 2–3 days (each session consisted of 20 min of stimulation)	Pre-post and up to 75 min after the end of each stimulation	MEPs in the biceps brachii, triceps brachii, and the first dorsal interosseous. CMEPs were elicited by simulation at the corticomedullary junction by using high-voltage electrical current at an intensity to generate CMEPs. SICI was elicited by paired stimuli and was calculated for biceps brachii.	The size of CMEPs but not MEPs increased in proximal and distal arm muscles for 75 min after TSCS, but not sham-TSCS, in control subjects and SCI participants. SICI increased at different time intervals after TSCS compared with baseline in control and SCI participants. Effects of carrier frequency: both CMEPs and MEPs amplitude increased after the TSCS without 5 kHz compared with baseline	The mean time to complete all functional tasks decreased after TSCS with and without 5 kHz compared with sham TSCS. The decrease in time was larger after TSCS with compared with TSCS without 5 kHz
Hofstoetter et al. (2020)	Cathodes: T11-T12, Anodes: lower abdomen	AC-TSCS	Charge‐balanced, symmetric, biphasic rectangular pulses of 1 ms at 50 Hz	Sub‐motor threshold intensity	NA	NA	Single 30‐minute session of TSCS	Before (A0), immediately after (A1), and 2 h after (A2) stimulation	Achilles clonus was elicited by brisk manual ankle dorsiflexion, and cutaneous‐input‐evoked spasms by stroking the foot sole with a blunt rod	The amplitude of the activity associated with clonus were significantly reduced in both post‐stimulation assessments. The median RMS values of the EMG produced in response to plantar stimulation were significantly reduced,as well	MAS score, clonus, and spasms were significantly reduced immediately after TSCS, and all spasticity measures were improved 2 h postintervention. No changes in the median walking speed was reported
Meyer et al. (2020)	Cathode: T11-T12, Anode: lower abdomen	AC-TSCS	Symmetric, biphasic rectangular pulses of 1 ms width per phase.	0.8–1.0 times motor threshold	NA	NA	Session 1:examination of ankle movement ankle, session 2: spinal reflex activity and walking performance.	With and without TSCS in each session	TA flexion reflex was elicited by applying monopolar electrical stimulation to the distal tibial nerve	Tonic 30-Hz TSCS did not alter the spinal reflex threshold nor the amplitude of the early reflex component but it significantly reduced the EMG-RMS of the late reflex component	Tonic TSCS at 30 Hz immediately improved maximum dorsiflexion in the more affected lower limb during the rhythmic ankle movement task. During walking:3 participants with the lowest as well as the one with the highest walking function scores showed positive stimulation effects, including increased maximum walking speed
Shapkova et al. (2020)	Cathode: T12, Anode: centrally on the abdomen	AC-TSCS	0.5 ms monophasic, frequency of TSCS for the main group was set to 1 (Group 1), 3 (Group 2), and 67 (Group 3) pulses/s	1.3–1.4 of motor threshold	EWT	NA	8 sessions of combined EWT and TSCS over the 2 weeks of training (total duration of walking in the exoskeleton: 250–300 min). All subjects received daily 40 min TSCS in the stationary (supine) position before the EWT session	Pre and post	H-reflex responses in the lateral gastrocnemius muscle of both legs, and SMEPs in the rectus femoris, biceps femoris, lateral gastrocnemius, and tibialis anterior in response to the electrical stimulus (0.5 ms) at T11-12	The amplitude of SMEPs increased after the EWT + TSCS.An increase of this Hmax/Mmax by more than 30% in 3 subjects with initially low ratio and a decrease in 3 subjects with an initially high ratio	EWT with TSCS significantly increased the foot loading forces, and Hauser Ambulation Index. Group 1 (stimulation at 1 pulse/s), had no improvements in the motor scale, while in Group 2 (3 pulses/s) and Group 3 (67 pulses/s), the proportion of individuals with improvements in the AIS motor scale was comparable (6/9 and 3/4, respectively). In Group 4 (EWT without SCES), a substantially smaller proportion of individuals showed improvements in the AIS motor and sensory scales
Zhang et al. (2020)	Cathode: one above (C3-4) and one below (C7-T1)], Anode: over the iliac crests.	AC-TSCS with a carrier frequency of 10 kHz	Monophasic, rectangular pulses with 1 ms duration at a frequency of 30 Hz, with a carrier frequency of 10 kHz	Adjusted based on the participant’s functional task performance	Task-specific hand training	NA	18 sessions (60 min/session) over the course of 8 weeks	Pre-post, and 3 months follow up	SMEPs recorded from trapezius, lateral deltoid, biceps brachii, triceps brachii, brachioradialis, extensor carpi ulnaris, flexor carpi radialis, and extensor carpi radialis in response to monophasic, rectangular, 2 Hz, 1 ms pulses with a 10 kHz carrier frequency) was delivered at C3-4 and C7-T1	At the post-intervention, the amplitude of sMEPs and integrated evoked potentials from muscles significantly increased, as compared to the baseline.	The total score of the GRASSP, Sensibility, and Prehension improved post-intervention and maintained during the 3 month follow-up. The bilateral handgrip force improved at post and follow-ups
Islam et al. (2021)	Cathode: T10-L1, Anodes: either side of the abdominal muscles or iliac crests	AC-TSCS	Pulse train of 12 pulses at 333.3 Hz with a total duration of 33 ms	Pulse train transspinal stimulation intensity was delivered at 0.95 multiples of soleus SMEPss threshold, ranging from 57 to 160 mA, and produced mild trunk extension across subjects.	Treadmill walking with the assistance of a robotic gait orthosis	NA	1 session	Without, during, and after transspinal stimulation	The soleus H-reflex was recorded in both subject groups under control conditions and following single-pulse transspinal stimulation.	Transspinal stimulation, when delivered before posterior tibial nerve stimulation, reduced H-reflex excitability throughout the walking cycle	The phase-dependent locomotor muscle activity in SCI individuals was replaced with tonic activity throughout the walking cycle
Pulverenti et al. (2021)	Cathode: T10-L1-2, Anodes:iliac crests	AC- TSCS	1-ms monophasic square-wave pulse	Stimuli were delivered at soleus SMEPs threshold intensity	Group 1 SCI received Transspinal-TMS, group 2 recieved TMS-Transspinal.Paired stimuli were delivered at the mid-stance phase based on foot switch signals placed on the leg targeted by TMS.	NA	BWS walking training with the Lokomat for 5 days/week, 1 h/day for 5 weeks (25.8 ± 4.8 sessions; mean ± SD) including 40-min of paired stimuli during BWS walking, and 20-min of walking training without stimulation	Pre-post	During standing with BWS, the soleus H-reflex and M-wave recruitment input–output curves were assembled by sending approximately 80 stimuli at a range of intensities to the posterior tibial nerve at 0.2 Hz. M waves and H-reflexes recorded during walking were normalized to the Mmax evoked 60 ms after the test stimuli	When H-reflexes were grouped based on the TMS-targeted leg, transspinal-TMS PAS and locomotor training improved reflex inhibition during the swing phase, while TMS-transspinal PAS and locomotor training improved excitation during the stance phase	NA
Zaaya et al. (2021)	Cathode: T12, Anodes: centrally on the abdomen	AC-TSCS	333 Hz with a pulse train consisting of 12 pulses with a total 33 ms duration	0.8–1.2 times the right soleus (SOL) SMEPs	BWS walking with a robotic gait orthosis system	NA	An average of 18 sessions (range: 17–20), 5 days/week for 1 h/day.	Pre-post	TA flexion reflex was evoked based on the foot switches placed on the ipsilateral foot, and were delivered randomly at different phases of the entire walking cycle with a pulse train of 26.5 ms total duration at 333 Hz via a bipolar bar electrode placed along the right sural nerve.SMEPs were recorded during both supine and BWS walking.	The long-latency TA flexion reflex was depressed in all phases of the walking cycle while spinal motor output based on SMEPs recruitment curves was increased	
Zhang Z. et al. (2021)	Active electrode (cathode): T9-T11 Reference electrode: right shoulder	DC-TSCS	NA	Increased in the 5-mA interval from 5 mA to the maximum value without discomfort.	5 Different combination techniques in session 1: T1: 20-Hz rTMS (brain) and cathode DC-TSCS (spine), T2: 20-Hz rTMS (brain) and 20-Hz square wave (spine), T3: iTBS (brain) and cathode DC-TSCS (spine), T4: iTBS (brain) and iTBS(spine),T5: sham rTMS (brain), and sham DC-TSCS (spine)	2 control stimulation:(1) rTMS (brain) and sham (spine) and (2) sham (brain) and DC-TSCS (spine). For sham stimulation, electrodes were placed same active stimulation, but the stimulator was turned after 30 s.	2 training sessions	Pre-post	The latency and amplitude of MEPs for lower leg	The amplitude of MEP in the left lower leg increased following different types of PAS. The latencies of MEP improved after the application of different PAS	LEMS of the left lower leg increased after all paired treatments
Adeel et al. (2022)	Active electrode (anode): T10, Reference electrode: right/left deltoid	DC-TSCS	Stimulation was delivered for 1,200 s, by fading in and out every 10 s with	An intensity of 2.5 mA and a current density of 0.071 mA/cm2 over 20 min	For rTMS-iTBS/DC-TSCS, transcranial output consisted of 2-s-duration bursts of 5 Hz (10 pulses/ burst) with an intertrain interval of 8 s, lasted for 192 s (totally 600 stimuli) along with 2.5 mA of DC-TSCS. The intensity of the rTMS intervention was set to 90% of the RMT. After each paired stimulation intervention, a 30 min of bicycling exercise	Sham stimulation: the same inverted coil and spinal electrodes provided a similar sound but no stimulation	3 sessions of stimulation (each lasting around 1 h with a gap of 1 week)	Pre-post	MEPs latency and amplitude of the right and left tibialis anterior were measured at RMT, and H-Reflex	MEPs latency: significant differences between before and after stimulation in both the rTMS-20 Hz/DC-TSCS and rTMS-iTBS/DC-TSCS interventions. MEPs amplitude: only the rTMS-20 Hz/tsDCS intervention protocol showed a significant difference. No significant difference in H-Reflex	rTMS-iTBS/ DC-TSCS intervention exhibited a significant increase in LEMS:(*p* = 0.038) but the rTMS-20 Hz/DC-TSCS intervention did not show a significant difference. MAS: The score did not significantly change in either intervention protocol
Pulverenti et al. (2022)	Cathode: T10 to L1-2, Anodes: iliac crests	AC-TSCS	1 ms monophasic square-wave pulse	At soleus motor threshold (SMEPs)	Group 1 SCI received Transspinal-TMS, group 2 recieved TMS-Transspinal.Paired stimuli were delivered at the mid-stance phase based on foot switch signals placed on the targeted leg by TMS.	NA	All participants received BWS walking 5 days/week, 1 h/day for 5 weeks including 40-min of paired stimuli during BWS walking, and 20-min of training without stimulation	Pre-post	TA flexion reflex was by pulse train of 30-ms duration (1-ms pulses at 300 Hz) was delivered to the medial arch or sural nerve at the lateral malleolus of the foot	Both the early and late TA flexion reflex remained unaltered after TMS-transspinal, however, they significantly decreased after transspinal-TMS	NA
Samejima et al. (2022)	Cathodes: C3-4, C6-7, T11, and L1 anodes: iliac crests	AC-TSCS With a carrier frequency of 10 kHz	Biphasic, rectangular, 1 ms pulses at a frequency of 30 Hz	Below the motor threshold	BWS walking		1.5 to 2 h/d, 3 to 4 times per week (TSCS for 1.5 to 2 h per session)	Every 1–2 months	Somatosensory evoked potentials: Stimulation of the tibial nerve and recorded cortical potentials between Cz’ and Fz	Shorter latencies of P40 following the TSCS phases of the study	3 times improvement in 6MWT along with improvements in balance (BBS), sensation, bowel and bladder function after TSCS phase

Activity-based therapy (ABT), alternating current (AC), American Spinal Injury Association Impairment Scale (AIS), Berg balance scale (BBS), body weight supported (BWS), cervicomedullary evoked potentials (CMEPS), direct current (DC), exoskeleton walking training (EWT), Graded and Redefined Assessment of Strength, Sensibility and Prehension (GRASSP), Hoffmann Reflex (H-Reflex), manual muscle testing (MMT), motor evoked potentials(MEPs), repetitive TMS (rTMS), resting motor threshold (RMT), 6-meter walk test (6MWT), short-interval intracortical inhibition (SICI), soleus (Sol), spinal cord injury (SCI), spinal cord independence measure (SCIM), spinal motor evoked potentials (SMEPs), 10-meter walk test (10MWT), tibialis anterior (TA), transcranial magnetic stimulation (TMS), transcutaneous spinal cord stimulation (TSCS), paired associative stimulation (PAS).

#### 3.2.1 Changes in the excitability of spinal pathways to the lower limb muscles following a single session of DC-TSCS

So far, only two studies have reported immediate changes in spinal excitability following single sessions of DC-TSCS in individuals with SCI. No studies have reported long-term effects of DC-TSCS on the spinal excitability in the SCI population. Hubli et al. (2013) measured spinal reflex amplitude and threshold by electrical stimulation of the tibial nerve posterior to the medial malleolus before and immediately after single sessions of training (20 min) across four experimental conditions: anodal DC-TSCS, cathodal DC-TSCS, sham DC-TSCS, and locomotion only. This was a case–control study including 17 individuals living with SCI [American Spinal Injury Association Impairment Scale (AIS): 10 AIS A, 7 AIS B], and 17 controls (Hubli et al., 2013). Findings showed that anodal DC-TSCS increased spinal reflex amplitude only in individuals with SCI while cathodal, sham, and locomotion did not affect the reflex amplitude. Furthermore, the reflex threshold decreased following anodal DC-TSCS and locomotor conditions only in individuals with SCI (Hubli et al., 2013). Adeel et al. (2022) measured H-reflex latency in response to tibial nerve stimulation in nine individuals living with chronic incomplete SCI (AIS B-D) before and immediately after single sessions of training across three paired stimulation conditions with a one-week gap period: repetitive TMS (rTMS at 20 Hz) with DC-TSCS for 1,200 s (intensity: 2.5 mA and a current density of 0.071 mA/cm^2^), and rTMS-iTBS (intermittent theta burst stimulation: iTBS) with anodal DC-TSCS and control (same coil over the vertex and same spinal electrodes on T10 with sound but no stimulation). Their findings indicate that the latencies of H-reflex did not change between the two active intervention protocols across nine individuals (Adeel et al., 2022).

#### 3.2.2 Changes in the excitability of spinal pathways to the lower limb muscles following a single session of AC-TSCS

Three studies reported immediate changes in the spinal pathways to the lower limb muscles following single sessions of AC-TSCS in individuals with SCI. Hofstoetter et al. (2020) measured stretch-induced spasticity, clonus, and cutaneous-input-evoked spasms before, immediately after, and 2 h after a single session of TSCS intervention (30 min at 50 Hz with sub-threshold intensity) in 12 individuals living with SCI (3 AIS A, 3 AIS C, 6 AIS D). Their findings showed reduced cutaneous-input-evoked spasms as measured by electromyography (EMG) activity in TA and soleus in response to mechanical stimulation as well as reduced spasticity and clonus at both post-stimulation assessments (Hofstoetter et al., 2020). Meyer et al. (2020). measured spinal reflexes elicited by applying monopolar electrical stimulation to the distal tibial nerve in a session with and without TSCS. They found reduced EMG amplitude of the late reflex component with tonic 30-Hz TSCS. Islam et al. applied TSCS in a single session (333.3 Hz at 0.95 motor threshold of soleus muscle) randomly across the step cycle and found that when TSCS was delivered before posterior tibial nerve stimulation during treadmill walking, it reduced H-reflex excitability across the step cycle in individuals with incomplete SCI (1 AIS B, 1 AIS C, 3 AIS D) (Islam et al., 2021).

#### 3.2.3 Changes in the excitability of spinal pathways to the lower limb muscles following multiple sessions of AC-TSCS

Eight studies reported changes in the spinal pathways to the lower limb muscles following multiple sessions of AC-TSCS. Gerasimenko et al. (2015) investigated the effects of multiple sessions of TSCS on spinal excitability measured by the peak-to-peak amplitude of SMEPs and recruitment curves from major lower limb muscles during T11 stimulation in the supine position (at rest and a plantarflexion effort) at the following periods: pre-training (baseline), post-training (after 4 weeks of TSCS in the side-lying position plus active & passive limb oscillation), pre-drug (10 weeks of maintenance of the same procedures but without the oscillation), and post-drug (TSCS plus buspirone 7.5 mg administered orally twice daily for the last 4 weeks) in five individuals with SCI (AIS B). They found that the amplitude of SMEPs in the lower limb muscles (medial hamstring, medial gastrocnemius and tibialis anterior) was higher at post-drug compared to pre-train (Gerasimenko et al., 2015). Although the amplitude of the early response (latency of about 25–30 msec) did not change during the plantarflexion effort at pre-train, there was a dramatic increase in the amplitude of the early response at post-drug (Gerasimenko et al., 2015). Furthermore, a late response latency of about 100–1,000 msec was present during both rest and the plantarflexion effort at the post-drug (Gerasimenko et al., 2015). Two other studies investigated the long-term effects of TSCS intervention protocol, i.e., multiple sessions of AC-TSCS over T10-L2 on soleus H-reflex excitability and SMEPs of lower limb muscles in 10 individuals with SCI (2 AIS A, 2 AIS B, 1 AIS C, 5 AIS D) and 10 healthy controls (Knikou and Murray, 2019; Murray and Knikou, 2019a). Knikou and Murray (2019) found that soleus H-reflex excitability decreased in individuals living with SCI but not in healthy controls. They also measured soleus H-reflex homosynaptic and post-activation depression by stimulating the posterior tibial nerve at different frequencies (0.1, 0.125, 0.2, 0.33, and 1.0 Hz) and paired tibial nerve stimuli at different intervals, respectively. Their findings showed increased homosynaptic depression after the intervention, however, post-activation depression remained unaltered after intervention (Knikou and Murray, 2019). Murray and Knikou (2019b) found that in the same study population, the amplitude of SMEPs increased for knee muscles in the controls and individuals with complete SCI, while in individuals with motor incomplete SCI, the amplitude of SMEPs increased for both ankle and knee muscles. Sayenko et al. (2019) found that the TSCS intensity required to reach the motor threshold in the leg muscles decreased across multiple sessions of balance training combined with TSCS (over the T11-L1) in six individuals living with complete SCI. Shapkova et al. (2020) measured H-reflex responses in the lateral gastrocnemius muscle of both legs, and SMEPs in the major lower limb muscles (rectus femoris, biceps femoris, lateral gastrocnemius, and tibialis anterior) in response to the electrical stimulation at T11-T12 following multiple sessions of combined exoskeleton walking training (EWT) and TSCS in a sample of 35 individuals living with SCI [group 1: 19 SCI (11 AIS A, 5 AIS B, 3 AIS D, group 2: 16 SCI (7 AIS A, 5 AIS C, 4 AIS D)]. They found that the amplitude of SMEPs increased after the EWT + TSCS (Shapkova et al., 2020). Regarding the Hmax/Mmax (maximal H-reflex relative to maximal M-wave), they found an increase in the ratio in six participants who had an initially low Hmax/Mmax, and a decrease in the ratio in three participants who had an initially high Hmax/Mmax ratio.

Two randomized controlled trials (RCTs) conducted by Pulverenti et al. looked at differences in the soleus H-reflex and TA flexion reflex immediately before and 1 day after the last training session (5 days/week for 30 sessions) between two groups of individuals with complete and incomplete SCI that received paired associative stimulation (PAS) (Pulverenti et al., 2021, 2022). Group 1 received transspinal-TMS combined with locomotor training (1 AIS A, 1 AIS B, 3 AIS C, 3 AIS D) while group 2 received TMS-transspinal combined locomotor training (1 AIS A, 3 AIS C, 2 AIS D) (Pulverenti et al., 2021). They found that when soleus H-reflexes were grouped based on the TMS-targeted limb, transspinal-TMS PAS increased reflex inhibition during the swing phase, while TMS-transspinal PAS increased reflex excitation during the stance phase (Pulverenti et al., 2021). Furthermore, both transspinal-TMS and TMS-transspinal PAS increased EMG amplitude and promoted a more physiological modulation of motor activity. Pulverenti et al. (2022) also found that the early and late TA flexion reflexes were significantly depressed during stepping in the group that received paired transspinal-TMS and locomotor training (1 AIS A, 2 AIS C, 2 AIS D) while remained unaltered in the group that received TMS-transspinal and locomotor training (1 AIS A, 3 AIS C, 2 AIS D). Zayaa et al. investigated the effects of 18 sessions of TSCS combined with body weight-supported (BWS) training on the long-latency TA flexion reflex and SMEPs in five individuals with SCI (AIS B). They found that the long-latency TA flexion reflex decreased across the step cycle while SMEPs based on recruitment curves increased after the intervention (Zaaya et al., 2020).

#### 3.2.4 Changes in the excitability of spinal pathways to the upper limb muscles following a single session of AC-TSCS

Benavides et al. (2020) investigated acute single-session effects of AC-TSCS (with and without carrier frequency) over the cervical spinal cord on spinal and corticospinal excitability and found that TSCS had an excitatory effect at the spinal level as measured by the size of CMEPs induced by high-voltage electrical current stimulation at cervicomedullary junction.

#### 3.2.5 Changes in the excitability of spinal pathways to the upper limb muscles following multiple sessions of AC-TSCS

Two studies reported the effects of multiple training sessions of AC-TSCS on the excitability of spinal pathways. Gad et al. (2018) measured SMEPs from proximal and distal upper extremity muscles by applying TSCS pre, during, and post-intervention (4 weeks of TSCS intervention combined with hand grip tasks) in six individuals living with SCI (2 AIS B, 4 AIS C). They found larger responses in the amplitude of SMEPs for distal muscles at the end of the intervention along with increased hand grip function compared with before the intervention (Gad et al., 2018). Inanici et al. (2018) also measured SMEPs over C3-C4 from a 62-year old male with C3, incomplete, chronic SCI (AIS D) following different periods of rehabilitation including multiple sessions of physical therapy with and without TSCS. They found that polysynaptic, late EMG responses increased gradually over 4 weeks of stimulation combined with physical therapy, reduced after physical therapy only, but returned with 5 days of additional stimulation and therapy treatment. Zhang et al. (2020) measured SMEPs response in the upper limb muscles following 18 sessions (60 min/session) over 8 weeks in a 38-year-old male with a C5 SCI (AIS A) and found that the amplitude of SMEPs and integrated evoked potentials from upper limb muscles significantly increased, as compared to the baseline.

### 3.3 Changes in the excitability of supraspinal pathways following TSCS

Seven studies measured corticospinal and intracortical excitability using TMS techniques. Of the included studies, five reported changes following DC-TSCS (Powell et al., 2016, 2018; Abualait and Ibrahim, 2020; Zhang et al., 2020; Adeel et al., 2022), and two studies reported changes following AC-TSCS (Murray and Knikou, 2017; Benavides et al., 2020). We did not discern between upper and lower extremity studies in the following session given the paucity of studies investigating TSCS for the upper extremity function. Results from MEPs are shown in Table 2 and are reported in detail below.

#### 3.3.1 Changes in the excitability of supraspinal pathways following a single session of DC-TSCS

Three studies measured the acute effects of a single training session with DC-TSCS on the excitability of supraspinal pathways. Powell et al. (2018) measured MEPs bilaterally from the soleus before and after the single-session intervention of DC-TSCS across 3 conditions including cathodal, anodal, and sham DC-TSCS in five subjects with chronic, incomplete SCI (3 AIS C, 2 AIS D). Although no significant difference in the MEPs amplitude was found between the three conditions, there was a trend toward laterality of MEPs responses with DC-TSCS, i.e., corticospinal excitability increased contralateral to the reference electrode and decreased ipsilateral to the reference electrode (Powell et al., 2018). Zhang Z. et al. (2021) combined repetitive TMS (rTMS) with TSCS in three individuals with incomplete SCI (2 AIS C, 1 AIS D) and looked at the acute single session effects of 5 paired stimulation conditions on the MEPs (Zhang Z. et al., 2021). They showed that 20-Hz rTMS combined with cathodal DC-TSCS had the greatest effect on corticospinal excitability as measured by the amplitude and latency of MEPs of the lower leg before and after the intervention (Zhang Z. et al., 2021). Adeel et al. investigated corticospinal excitability following single sessions of three paired stimulation interventions with a 1-week gap period: control, repetitive TMS (rTMS) at 20 Hz with anodal DC-TSCS, and iTBS with DC-TSCS. For rTMS-iTBS/DC-TSCS, TMS was delivered in 2-s-duration bursts of 5 Hz (10 pulses/ burst) with an interval of 8 s along with 2.5 mA of DC-TSCS (Adeel et al., 2022). They found that the MEPs latency decreased and the amplitude increased with the rTMS-iTBS/DC-TSCS or the rTMS-20 Hz/DC-TSCS protocols compared to the control intervention (Adeel et al., 2022).

#### 3.3.2 Changes in the excitability of supraspinal pathways following multiple sessions of DC-TSCS

Two studies reported the effects of multiple training sessions with DC-TSCS on the excitability of supraspinal pathways. Powell et al. (2016) measured corticospinal excitability in a cross-over study following 24 sessions of cathodal DC-TSCS and 24 sessions of sham DC-TSCS paired with locomotor training on a robotic gait orthosis in a single subject with motor incomplete SCI (AIS D). They found that MEPs of soleus muscle increased following cathodal DC-TSCS but not sham DC-TSCS (Powell et al., 2016). Abualait and Ibrahim (2020) investigated changes in the MEPs after many sessions of intervention with DC-TSCS (30 sessions of cathodal DC-TSCS and 30 sessions of anodal DC-TSCS intervention) and found that in two patients with incomplete SCI (2 AIS C), MEPs increased in the post-cathode and deteriorated in the post-anode.

#### 3.3.3 Changes in the excitability of supraspinal pathways following a single session of AC-TSCS

Benavides et al. also examined cortical MEPs in arm muscles pre and post single sessions of intervention with and without TSCS (20 min of TSCS with 30 Hz and a 5 kHz carrier frequency and sham-TSCS) in individuals with and without chronic incomplete cervical SCI (Benavides et al., 2020). They found that the amplitude of MEPs increased in proximal and distal arm muscles in both SCI and healthy control groups when TSCS was applied without the 5 kHz carrier frequency (Benavides et al., 2020). Intracortical inhibition evoked by paired stimuli increased after TSCS in both SCI and control groups.

#### 3.3.4 Changes in the excitability of supraspinal pathways following multiple sessions of AC-TSCS

Murray et al. measured cortical and corticospinal excitability with paired and single pulses, respectively in an individual with motor incomplete SCI (AIS C for upper extremities & AIS B for lower extremities) following multiple sessions (15 sessions) of daily TSCS (an average of 55 min). They found an increase of MEPs in response to paired TMS pulses (intracortical facilitation) immediately after training in wrist flexor and extensor muscles, recovered intracortical inhibition (decrease in MEPs in response to paired TMS pulses) in the more impaired wrist flexor muscle, and increased corticospinal excitability bilaterally (Murray and Knikou, 2017).

### 3.4 Changes in the somatosensory evoked potentials following multiple sessions of AC-TSCS

Samejima et al. (2022) measured somatosensory evoked potentials (SSEPs) by stimulation of the tibial nerve posterior to the medial malleolus and recorded cortical potential following 2 months of intensive locomotor training and 2 months of multisite cervical and lumbosacral AC-TSCS paired with intensive locomotor training in two individuals living with incomplete SCI (2 AIS D). They found an improvement in P40 latencies of the tibial SSEPs following the TSCS phases of the study (Samejima et al., 2022).

### 3.5 Training modality and dosage

Of the included studies, 15 utilized AC-TSCS only, 4 utilized DC-TSCS only, and 4 paired spinal stimulations with other types of stimulations, i.e., mainly rTMS. Among the studies that utilized AC-TSCS, the majority used a burst frequency of 0.2–30 Hz while seven used TSCS with carrier frequency (6 used 10 kHz and 1 used 5 kHz). Two studies applied TSCS over the thoracolumbar region at a frequency of 333 Hz during robotic-assisted step training (Islam et al., 2021; Zaaya et al., 2021). Hofstoetter et al. (2020) applied TSCS at a frequency of 50 Hz to attenuate spasticity. Of the included studies that utilized AC-TSCS, eight applied a monophasic waveform (Gerasimenko et al., 2015; Knikou and Murray, 2019; Murray and Knikou, 2019b; Sayenko et al., 2019; Shapkova et al., 2020; Zhang et al., 2020; Pulverenti et al., 2021, 2022), five applied a biphasic waveform (Inanici et al., 2018; Benavides et al., 2020; Hofstoetter et al., 2020; Meyer et al., 2020; Samejima et al., 2022), one reported both mono- and biphasic waveforms (Gad et al., 2018), and three did not report the type of waveform used (Murray and Knikou, 2017; Islam et al., 2021; Zaaya et al., 2021). Regarding the pulse width, 12 studies used 1 ms pulse width (Gerasimenko et al., 2015; Gad et al., 2018; Knikou and Murray, 2019; Murray and Knikou, 2019b; Sayenko et al., 2019; Hofstoetter et al., 2020; Meyer et al., 2020; Zhang et al., 2020; Pulverenti et al., 2021, 2022; Samejima et al., 2022), two studies used 0.2 ms (Benavides et al., 2020) and 0.5 ms pulse width (Shapkova et al., 2020), and three did not specify the used pulse width (Murray and Knikou, 2017; Islam et al., 2021; Zaaya et al., 2021). Sixteen studies used TSCS in combination with other physical interventions such as robot-assisted gait training, activity-based physical therapy, and visual feedback training while seven used stimulation techniques only. Eight studies looked at the acute effects of TSCS on neural excitability following a single session of training (Hubli et al., 2013; Powell et al., 2018; Benavides et al., 2020; Hofstoetter et al., 2020; Meyer et al., 2020; Islam et al., 2021; Zhang H. et al., 2021; Adeel et al., 2022) while 15 studies investigated the effects of multiple sessions of TSCS on the excitability of neural pathways (Gerasimenko et al., 2015; Powell et al., 2016; Murray and Knikou, 2017; Gad et al., 2018; Inanici et al., 2018; Knikou and Murray, 2019; Murray and Knikou, 2019a; Sayenko et al., 2019; Abualait and Ibrahim, 2020; Shapkova et al., 2020; Zhang et al., 2020; Pulverenti et al., 2021; Zaaya et al., 2021; Pulverenti et al., 2022; Samejima et al., 2022). Total training time across studies varied from a single session to 4–5 sessions per week for a maximum duration of 9 weeks. The duration of the sessions varied between 5 and 120 min.

### 3.6 Quality assessment

The quality of the included studies was assessed using the D&B Checklist (Supplementary material). The overall quality was poor (mean score: 11.04 ± 1.55) with results ranging from 8 to 14. None of the included studies had a course of follow-up to measure neurophysiological outcomes after the cessation of intervention. Concerning blinding, eight studies reported that subjects were blinded to the interventions that they received (Hubli et al., 2013; Powell et al., 2016, 2018; Sayenko et al., 2019; Abualait and Ibrahim, 2020; Benavides et al., 2020; Pulverenti et al., 2021, 2022), however, in only three studies assessors were blinded to the intervention (Powell et al., 2016; Sayenko et al., 2019; Abualait and Ibrahim, 2020). With regard to randomization, subjects were randomized to different intervention groups/conditions in eight studies (Inanici et al., 2018; Powell et al., 2018; Sayenko et al., 2019; Benavides et al., 2020; Meyer et al., 2020; Pulverenti et al., 2021, 2022; Adeel et al., 2022). Moreover, eight studies used a placebo intervention in the form of sham stimulation (Hubli et al., 2013; Powell et al., 2016,^43,53^; Abualait and Ibrahim, 2020; Benavides et al., 2020; Zhang Z. et al., 2021; Adeel et al., 2022).

## 4 Discussion

### 4.1 Summary of findings

The current review is the first to systematically investigate changes in the excitability of neural pathways following different types of TSCS currents in individuals living with SCI. We should acknowledge that meta-analysis was precluded in our study due to the heterogeneity of study designs (case studies, case series, case–control, single arm pre-post and RCT), diversity of patient populations (different inclusion criteria), different TSCS intervention settings (type of stimulation, location, intensity, etc.) and number and variability of outcomes measured (MEPs, SMEPs, H-reflex, etc). Therefore, the results presented in this systematic review should be interpreted with caution given the absence of a meta-analysis to indicate statistical significance. Even though the quality of the included studies was low (D&B ≤ 14), there seems to be a trend showing that TSCS either alone or in combination with other physical interventions such as robot-assisted gait training, activity-based physical therapy, and visual feedback training can augment neuroplasticity by modulating the excitability of spinal and supraspinal neuronal networks in individuals living with SCI (Powell et al., 2016, 2018; Sayenko et al., 2019; Shapkova et al., 2020; Zhang et al., 2020; Pulverenti et al., 2021, 2022; Zaaya et al., 2021; Samejima et al., 2022). Most studies included in this systematic review also reported an improvement in motor performance as measured by clinician-based and performance-based tests (Gerasimenko et al., 2015; Gad et al., 2018; Inanici et al., 2018; Abualait and Ibrahim, 2020; Benavides et al., 2020; Zhang et al., 2020; Islam et al., 2021; Zaaya et al., 2021; Zhang Z. et al., 2021; Adeel et al., 2022). Regarding the effects of TSCS on spinal excitability, most studies showed that the amplitude of SMEPs consistently increased following multiple sessions of AC-TSCS (Gad et al., 2018; Murray and Knikou, 2019b; Sayenko et al., 2019; Zhang et al., 2020). However, a decrease in the amplitude of soleus H-reflex and long latency flexion reflex have been reported immediately after a single session of TSCS (Hofstoetter et al., 2020; Meyer et al., 2020; Islam et al., 2021), and after a period of training (Knikou and Murray, 2019; Shapkova et al., 2020; Zaaya et al., 2021). Regarding corticospinal excitability, studies showed that MEPs increased both following single and multiple sessions of cathodal DC-TSCS (Powell et al., 2016, 2018; Abualait and Ibrahim, 2020; Benavides et al., 2020; Adeel et al., 2022). In addition, the immediate effect of single sessions of PAS of the brain (rTMS) and spine (DC-TSCS) was more effective than only brain stimulation or DC-TSCS for improving corticospinal excitability (Zhang et al., 2020; Adeel et al., 2022). Nonetheless, it is difficult to conclude whether the neuroplasticity induced in the spinal and supraspinal pathways can be preserved as there was no long-term follow-up after the cessation of intervention in any of the included studies.

### 4.2 Changes in the excitability of spinal pathways following single and multiple sessions of TSCS

Spinal plasticity was reported in 16 studies including any of the following outcomes: soleus H-reflex (Knikou and Murray, 2019; Shapkova et al., 2020; Pulverenti et al., 2021; Adeel et al., 2022), TA flexion reflex (Hubli et al., 2013; Meyer et al., 2020; Zaaya et al., 2021; Pulverenti et al., 2022), SMEPs induced by pulse TSCS (Gerasimenko et al., 2015; Gad et al., 2018; Inanici et al., 2018; Murray and Knikou, 2019b; Sayenko et al., 2019; Shapkova et al., 2020; Zhang et al., 2020; Zaaya et al., 2021), CMEPs induced by electrical stimulation at the cervicomedullary junction (Benavides et al., 2020), and cutaneous-input-evoked response induced by stroking the foot sole with a blunt rod (Hofstoetter et al., 2020). Current neurophysiological findings indicate a decrease in monosynaptic spinal reflex excitability (soleus H-reflex excitability) and TA flexion reflex excitability following single (Hofstoetter et al., 2020; Meyer et al., 2020; Islam et al., 2021) and multiple sessions of AC-TSCS (Knikou and Murray, 2019; Shapkova et al., 2020; Zaaya et al., 2021) which was mainly associated with decreased spasticity and clonus. Although there is considerable variation in the methodology including the designs of studies, settings of training, and the parameters of TSCS stimulation, it appears that reduced spinal excitability as measured by peripheral nerve stimulation techniques (H-reflex, TA flexion reflex) after intervention with TSCS correlates with decreased spasticity. It has been frequently discussed in the literature that reduced presynaptic inhibition after SCI contributes to the exaggerated stretch reflexes associated with spasticity (Faist et al., 1994; Grey et al., 2008; Alashram et al., 2023). Therefore, it is probable that continuous generation of afferent activities in multiple roots by TSCS results in synchronous neurotransmitter release from the Ia terminals eventually leading to a prolonged decrease of neurotransmitter release, facilitation of presynaptic inhibition, and post-activation depression (Faist et al., 1994; Aymard et al., 2000; Hofstoetter et al., 2020; Alashram et al., 2023). This will reduce the facilitation of persistent inward currents (Heckman et al., 2008; ElBasiouny et al., 2010), and eventually regulate muscle tone (Knikou and Murray, 2019; Hofstoetter et al., 2020; Meyer et al., 2020; Shapkova et al., 2020; Alashram et al., 2023). However, to consolidate these findings, further high-quality studies with a large sample size that investigate both neurophysiological and clinical outcomes following TSCS intervention post-SCI are strongly warranted.

Over the last two decades, a growing number of studies have investigated changes in the excitability of neural pathways as a result of PAS including peripheral nerve stimulation (PNS) and motor cortex stimulation in humans (di Lazzaro et al., 2009; Knikou, 2017). In the SCI population, the majority of studies have also applied classical PAS of PNS-TMS during a non-functional resting state to improve hand motor function and neuroplasticity after SCI (Tolmacheva et al., 2017, 2019; Rodionov et al., 2019). Pairing TMS with TSCS during a motor activity (e.g., locomotor training) is a novel neuromodulation method to promote neuroplasticity post-SCI (Knikou, 2017; Pulverenti et al., 2021, 2022). Of the included studies in our review, two single-blind RCTs reported neurophysiological changes following pairing TMS-TSCS in individuals with SCI. Their findings showed that based on the TMS-targeted limb, PAS can have different effects, i.e., TSCS-TMS can increase H-reflex inhibition during the swing phase, While TMS-TSCS can increase H-reflex excitation during the stance phase (Pulverenti et al., 2021, 2022). Even though these studies provide evidence of the neuroplasticity in the spinal circuitries following paired spinal and brain stimulation over multiple sessions of training, no clinical measures were reported to further investigate whether neurophysiological changes induced by PAS can result in greater functional improvement (Pulverenti et al., 2021, 2022). In addition, there were no control experiments to measure whether the induced changes following PAS were superior to brain or spinal stimulation alone (Pulverenti et al., 2021, 2022). More human studies with rigorous design are needed to investigate and compare the effects of combined neuromodulation (pairing TSCS with other techniques such as TMS, and functional electrical stimulation) versus single neuromodulation technique on the functional and neural recovery post-SCI.

Regarding SMEPs induced by pulse TSCS, the included studies showed the amplitude of SMEPs of multiple motor neurons innervating the upper and lower limb muscles increased along with an increase in voluntary EMG and sensorimotor function after multiple sessions of AC-TSCS (Gerasimenko et al., 2015; Gad et al., 2018; Inanici et al., 2018; Murray and Knikou, 2019b; Sayenko et al., 2019; Shapkova et al., 2020; Zaaya et al., 2020, 2021). One case–control study showed that a single session of AC-TSCS, with and without carrier frequency, had an excitatory effect at the spinal level as measured by CMEPs in both individuals living with chronic incomplete SCI and healthy controls (Benavides et al., 2020). Indeed, a recent study that combined biophysical modeling with animal and human (individuals with SCI and stroke) electrophysiological experiments indicated that in the presence of supraspinal inputs, subthreshold excitatory postsynaptic potentials induced by spinal stimulation can be transformed into action potentials that increase motor output (Balaguer et al., 2023). Although a more detailed understanding of the neural mechanisms associated with spinal stimulation comes from preclinical and neurophysiological studies with epidural spinal cord stimulation, we believe that TSCS can also re-activate functionally silent pathways by enhancing the general level of excitability and bringing interneurons and motor neurons closer to the threshold of firing, thereby making the spinal circuits more likely to respond to both descending drives and ascending sensory information (Taylor et al., 2021; Barss et al., 2022; Lin et al., 2022; Sayenko et al., 2022).

Differences in the SMEPs (increase) compared to H-reflex (decrease) in response to intervention with TSCS may be due to differences in the neural pathways involved during testing, i.e., each single TSCS pulse can simultaneously stimulate dorsal root afferent fibers or other neuronal structures (e.g., neuronal cell bodies, glial cells), opening the potential for heteronymous inputs upon the multiple motoneuron pools while H-reflex is elicited mainly by stimulation of afferents in the peripheral nerve (Minassian et al., 2007; Moon et al., 2021). Evidence related to the neuromodulatory effects of DC-TSCS on spinal excitability is limited in this review. Only 2 studies have reported immediate changes in the spinal excitability following a single training session (H-reflex and TA flexion reflex) following DC-TSCS (Hubli et al., 2013; Adeel et al., 2022). Hubli et al. (2013) reported an increase in TA flexion reflex amplitude after a session of anodal DC-TSCS while Adeel et al. found the latencies of soleus H-reflex did not change in response to PAS of brain (rTMS) and anodal DC-TSCS (Adeel et al., 2022). Further studies are needed to enable a better understanding of the long-term effects of cathodal and anodal DC-TSCS on spinal excitability in individuals living with SCI.

### 4.3 Changes in the excitability of supraspinal pathways following TSCS

Changes in the excitability of corticospinal pathways related to lower limb muscles (MEPs of lower leg muscles induced by TMS) were mainly reported following DC-TSCS or PAS of the brain and spine (DC-TSCS) (Powell et al., 2016, 2018; Abualait and Ibrahim, 2020; Zhang et al., 2021; Adeel et al., 2022). Findings from these studies demonstrated an increase in the amplitude of MEPs linked to lower limb muscles following single sessions of cathodal DC-TSCS or PAS involving rTMS of the brain and DC-TSCS on the spine (Powell et al., 2016, 2018; Abualait and Ibrahim, 2020; Zhang et al., 2021). Only two studies investigated differences between cathodal and anodal DC-TSCS on corticospinal excitability (Powell et al., 2018; Abualait and Ibrahim, 2020). Powell et al. (2018) found no significant differences in the change of MEPs amplitude following single sessions of anodal, cathodal, and sham stimulation in five individuals with incomplete SCI. However, Abulait et al. found that MEPs induced by TMS only increased after many sessions of cathodal DC-TSCS combined with walking (robot-assisted gait training) and deteriorated after anodal DC-TSCS in two individuals with incomplete SCI (Abualait and Ibrahim, 2020). So far, Several studies in neurologically intact individuals have reported polarity-specific changes in the corticospinal excitability induced by DC-TSCS such that cathodal DC-TSCS can increase corticospinal excitability and decrease spinal reflexes while anodal DC-TSCS appears to decrease corticospinal excitability and increase spinal reflexes (Bocci et al., 2015; Schweizer et al., 2017; Powell et al., 2018). We believe that more studies are required to investigate polarity-specific differences in the neuroplasticity and functional outcomes by induced DC-TSCS in individuals living with SCI.

Regarding changes in the excitability of corticospinal pathways related to the upper limb muscles, so far only 2 studies have investigated changes in the MEPs associated with upper limb muscles following TSCS. Findings revealed that single session (Benavides et al., 2020) or multiple sessions (Murray and Knikou, 2017) of AC-TSCS without carrier frequency can increase the MEPs amplitudes associated with upper extremity muscles along with improvement in voluntary muscle strength and upper limb motor function (Murray and Knikou, 2017; Benavides et al., 2020). We believe that further understanding of the corticospinal mechanisms associated with recovery of upper limb motor function following TSCS intervention specifically targeted at the cervical enlargement is of significant importance after SCI as cervical spinal motorneurons receive extensive inputs from corticospinal tracts (Balbinot et al., 2023). Emerging evidence also suggests that the neuroplasticity induced by spinal cord stimulation also depends on the number of residual supraspinal inputs survived, thereby limiting the effects of TSCS in situations involving a substantial loss of supraspinal axons (Balaguer et al., 2023). Future studies should subgroup participants based on the location and severity of the lesion to further investigate whether neuroplasticity-induced changes following TSCS vary among different subgroups with SCI.

So far, there have been some reports indicating that different TSCS settings (waveform, frequency, amplitude) can have a different impact on motor recovery. Several of the included studies in our review have used AC-TSCS with 1 ms pulse width to improve motor function. Rehman et al. (2023) also recommended the use of a 1 ms pulse width to decrease pain and improve motor function. However, no studies have yet compared whether different pulse widths can alter the recruitment of neurons differently. Regarding the frequency, two studies reported the effects of different frequencies on motor recovery (Shapkova et al., 2020; Sayenko et al., 2022). Shapkova et al. (2020) compared the effects of different frequencies of 1 Hz, 3 Hz, and 67 Hz and reported that the application of 67 Hz had the greatest impact on spasticity and walking performance. Sayenko et al. (2019) applied different frequencies (5 Hz, 15 Hz, 25 Hz, and 30 Hz) and found that 15 Hz had the greatest effect on standing balance while 30 Hz was more effective in facilitating rhythmic stepping movements. However, none of the above-mentioned studies have discussed whether different frequencies or carrier frequencies have a differential impact on neural excitability. Benavides et al. were the first to compare the immediate single-session effects of carrier frequency on spinal and corticospinal excitability and found that both spinal and corticospinal excitability were facilitated when TSCS was applied without carrier frequency (Benavides et al., 2020). However, this study only evaluated motor and neural recovery outcomes up to 75 min after a single session of training. We believe that further exploration is warranted to characterize the impact of frequency and pulse width on modulating the excitability of neural pathways.

It should be acknowledged that the quality of all included studies ranged from eight to 14 indicating poor quality mainly due to lack of sufficient power, external and internal validity. Most of our reviewed studies received low scores due to lack of blinding, randomization, follow-up, inadequate power, study subjects not being representativeness of the entire population, lack of description of the confounders (age, sex, level, and severity of injury) and not having adequate adjustment for confounding factors in the analysis. Indeed, most of our findings were based on the results extracted from case studies, case series, and single group pre-post designs. Only two of our included studies conducted a RCT design to measure neurophysiological changes following PAS after SCI (Pulverenti et al., 2021, 2022). We believe that the quality of future studies would be improved by conducting larger sample or multi-site RCTs that allow for a greater number of participants to be recruited and can compensate for the high dropout rate that is commonly reported in the clinical trials in the SCI population. Studies should also consider an appropriate length of follow-up to measure any long-term neuroplastic changes that could be induced should following TSCS. Moreover, we suggest that future studies better identify the source population, improve the recruitment methods, and standardize the intervention protocols based on the currently available evidence.

In conclusion, the findings of this systematic review indicate that both DC and AC types of TSCS currents may augment neuroplasticity by modulating the excitability of spinal and supraspinal neuronal networks in individuals living with SCI. Specifically, findings showed an increase in the responsiveness of motoneuron pools over multiple spinal segments as measured by SMEPs following multiple sessions of AC-TSCS which contributed to better motor output. Reduction in the monosynaptic spinal reflex excitability (soleus H-reflex excitability) and flexion reflex arc was mainly reported after single and multiple sessions of AC-TSCS. The majority of studies showed the ability of cathodal DC-TSCS to modulate corticospinal excitability as shown by the increase in MEPs induced by TMS. We found a knowledge gap indicating a paucity of studies assessing cervical TSCS and its effects on the excitability of spinal and supraspinal neuronal networks to enhance upper extremity function in individuals living with SCI. Further high-quality clinical studies with larger samples investigating both clinical and neurophysiological measures in people with SCI may provide insight into the mechanisms occurring when a clinically meaningful improvement is obtained.

## Data availability statement

The original contributions presented in the study are included in the article/Supplementary material, further inquiries can be directed to the corresponding author.

## Author contributions

ST: Conceptualization, Formal analysis, Funding acquisition, Investigation, Methodology, Software, Writing – original draft, Writing – review & editing. GB: Conceptualization, Formal analysis, Investigation, Methodology, Writing – review & editing. MP: Investigation, Methodology, Writing – review & editing. DS: Conceptualization, Supervision, Writing – review & editing. JZ: Conceptualization, Formal analysis, Investigation, Methodology, Supervision, Writing – review & editing. KM: Conceptualization, Funding acquisition, Investigation, Methodology, Resources, Supervision, Writing – review & editing.

## Footnotes

^1^https://www.crd.york.ac.uk/prospero/display_record.php?RecordID=358829
